# Supplementary material for: LiFE, a multimodal circadian intervention, improves sleep, glycemic control, and recognition memory
Source: bioRxiv. 2026 Mar 16:2026.03.12.711428. Preprint. [Version 1] doi: 10.64898/2026.03.12.711428 (PMC13015279; doi:10.64898/2026.03.12.711428)
Supplement: Supplement 1 [file NIHPP2026.03.12.711428v1-supplement-1.pdf]

bioRxiv preprint doi: https://doi.org/10.64898/2026.03.12.711428; this version posted March 16, 2026. The copyright holder for this preprint (which was not certified by peer review) is the author/funder, who has granted bioRxiv a license to display the preprint in perpetuity. It is made available under aCC-BY 4.0 International license.

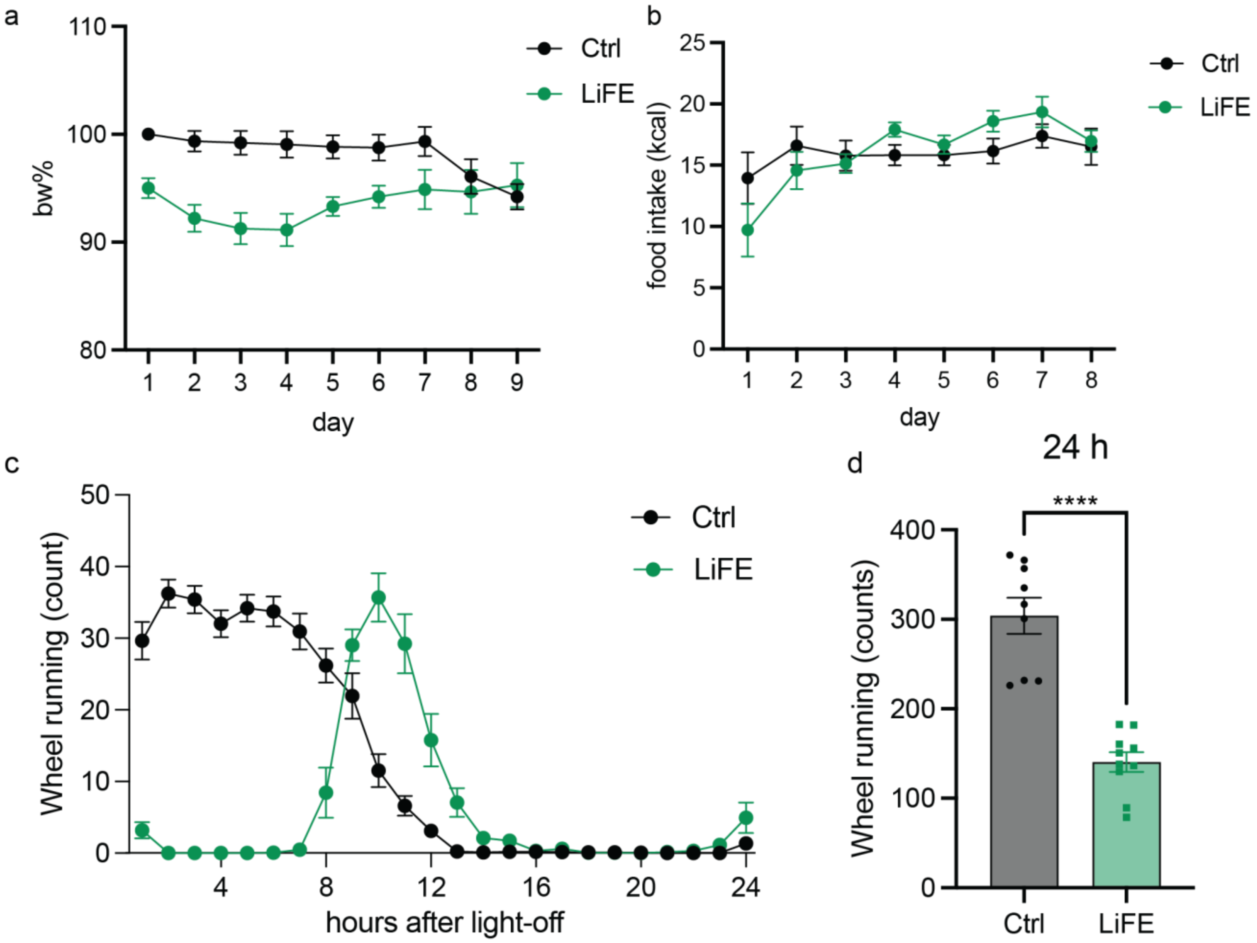

Figure S1. Characterizing behavior of WT mice in LiFE treatment.

a) Bodyweight of WT mice on control (n = 10) and LiFE (n = 16) schedules normalized to the control weight of day 1. **b)** Food consumption in every 24 hours measured with WT mice (n =6) on control and LiFE schedule. **c)** Wheel running activity in a 1 hour bin of WT mice under control (n = 9) and LiFE (n = 10) schedule. **d)** Total wheel running counts of data in a) (Student's t-test, ****P<0.0001).

bioRxiv preprint doi: https://doi.org/10.64898/2026.03.12.711428; this version posted March 16, 2026. The copyright holder for this preprint (which was not certified by peer review) is the author/funder, who has granted bioRxiv a license to display the preprint in perpetuity. It is made available under aCC-BY 4.0 International license.

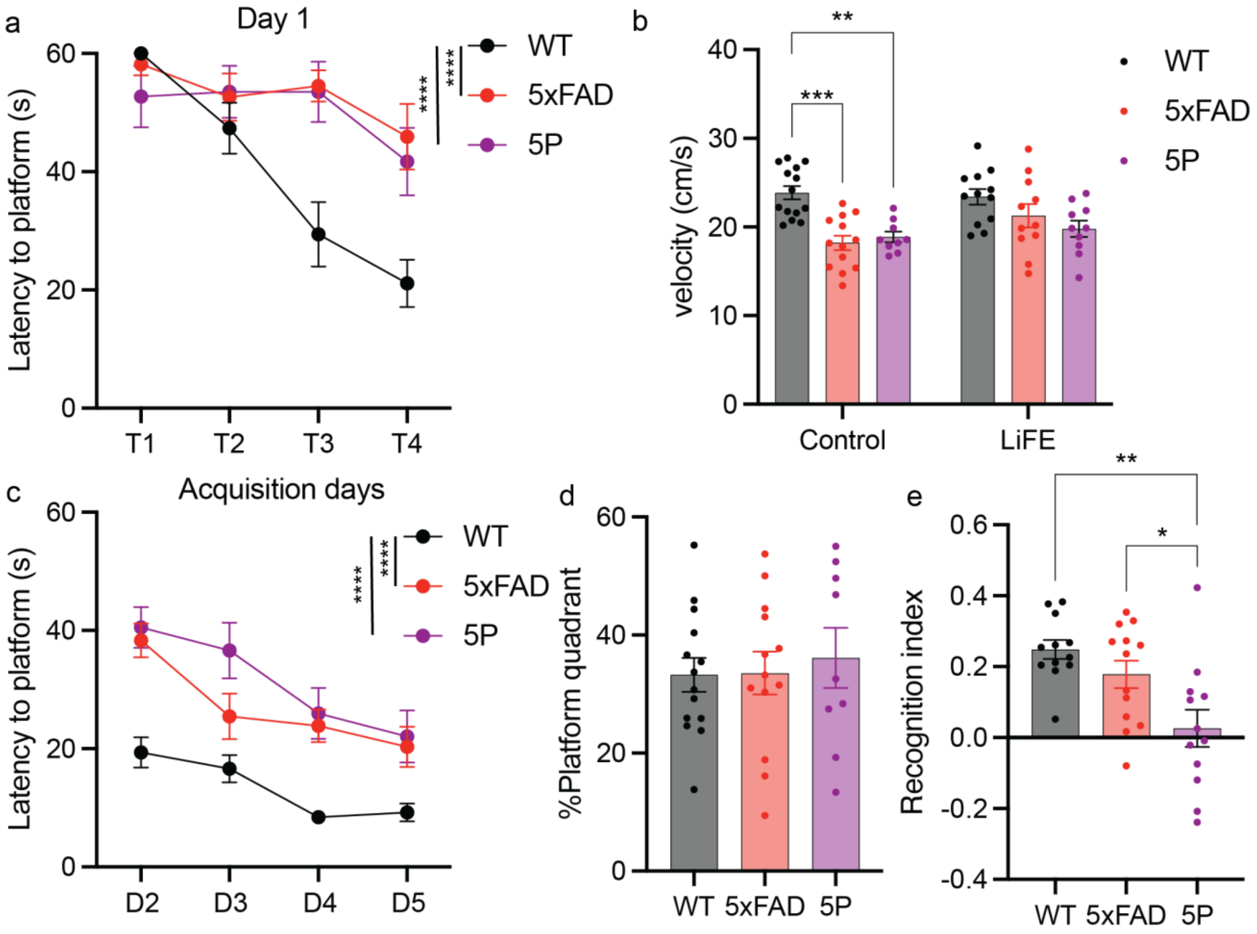

Figure S2. Comparison of cognitive behavior in WT and Alzheimer's mouse models.

a) Latency to the platform of WT (n = 14), 5xFAD (n = 13) and 5P (n = 9) control mice on day 1 learning in MWM (mixed-effect model with Bonferroni correction, ****P<0.0001). **b)** Swim speed of WT (control n = 14, LiFE n = 12), 5xFAD (control n = 13, LiFE n = 11) and 5P (control n = 9, LiFE n = 10) mice in MWM test in control and LiFE treatment group (two-way ANOVA with Bonferroni correction, **P<0.01, ***P<0.001).**c)** Latency to the platform of WT, 5xFAD and 5P control mice on acquisition days in MWM (mixed-effect model with Bonferroni correction, ****P<0.0001). **d)** Time-spent in target quadrant of WT, 5xFAD and 5P control mice on probe day in MWM. **e)** Recognition index of WT (n = 12), 5xFAD (n = 13) and 5P (n = 12) control mice in NOR (one-way ANOVA with Bonferroni correction, *P<0.05, **P<0.01).
